# Elevated Levels of Neutrophil-to Monocyte Ratio Are Associated with the Initiation of Paroxysmal Documented Atrial Fibrillation in the First Two Months after Heart Transplantation: A Uni-Institutional Retrospective Study

**DOI:** 10.3390/jcdd10020081

**Published:** 2023-02-15

**Authors:** Dragos-Florin Baba, Horatiu Suciu, Calin Avram, Manuela Gyorgy, Alina Danilesco, Laurentiu Huma, Ileana Anca Sin

**Affiliations:** 1 Emergency Institute for Cardiovascular Diseases and Transplantation, 540142 Targu Mures, Romania; 2 Department of Cell and Molecular Biology, George Emil Palade University of Medicine, Pharmacy, Science, and Technology of Targu Mures, 540142 Targu Mures, Romania; 3 Department of Surgery, George Emil Palade University of Medicine, Pharmacy, Science, and Technology of Targu Mures, 540142 Targu Mures, Romania; 4 Department of Medical Informatics and Biostatistics, George Emil Palade University of Medicine, Pharmacy, Science, and Technology of Targu Mures, 540142 Targu Mures, Romania; 5 Department of Psychology, Dimitrie Cantemir University, 999032 Bucharest, Romania

**Keywords:** neutrophil-to-monocyte ratio, monocyte-to-white cells ratio, inflammation, atrial fibrillation, heart transplant

## Abstract

Background: Heart transplantation represents the treatment for patients with end-stage heart failure (HF) being symptomatic despite optimal medical therapy. We investigated the role of NMR (neutrophil-to-monocyte ratio), NLR (neutrophil-to-lymphocyte ratio), NPR (neutrophil-to-platelet ratio), NWR (neutrophil-to-white cells ratio), MLR (monocyte-to-lymphocyte ratio), PLR (platelet-to-lymphocyte ratio), MWR (neutrophil-to-white cells ratio), and LWR (lymphocyte-to-white cells ratio) at the same cut-off values previously studied, to predict complications after heart transplant within 2 months after surgery. Methods: From May 2014 to January 2021, was included 38 patients in our study from the Cardiovascular and Transplant Emergency Institute of Târgu Mureș. Results: Preoperative NMR > 8.9 (OR: 70.71, 95% CI: 3.39–1473.64; *p* = 0.006) was a risk factor for the apparition of post-operative paroxysmal atrial fibrillation (Afib). In contrast, preoperative MWR > 0.09 (OR: 0.04, 95% CI: 0.003–0.58; *p* = 0.0182) represented a protective factor against AFib, but being the risk of complications of any cause (OR: 14.74, 95% CI: 1.05–206.59, *p* = 0.0458). Conclusion: Preoperative elevated levels of NMR were associated with the apparition of documented AFib, with high levels of MWR as a protective factor. High MWR was a risk factor in developing complications of any cause in the first 2 months after heart transplantation.

## 1. Introduction

Heart transplantation represents the treatment for patients with end-stage heart failure (HF) who remain symptomatic despite optimal medical therapy. The proportion of eligible donors is insufficient to meet the demand. Eligibility for cardiac transplantation implies a subjective evaluation focused on resting hemodynamic data and NYHA classification, the latter being frequently inaccurate, depending on various factors [1]. The first human-to-human heart transplant in history was performed on 3 December 1967, with a favorable immediate postoperative period. In later monitoring, thoracic radiography showed lung infiltrates and was mistakenly interpreted as part of graft rejection. After 18 days, the patient died secondary to severe pneumonia as a result of the intense immunosuppressive therapy [2]. Leading criteria for heart transplantation in adult patients are constituted by nonischemic and ischemic cardiomyopathy, followed by adult congenital heart diseases, valvular heart disease, and heart transplantation rejection, with an increasing proportion of female recipients [3].

Patient management once heart transplantation occurs represents a challenge following the complications that are attributed to the immunosuppressive therapy and graft rejection. Complications such as primary graft dysfunction, rejection, or infection can occur in early phases after heart transplantation, with one-year graft survival after transplantation of 74.2%. Additional life-threatening early-phase postoperative complications are related to procedural aspects and neurological and/or gastrointestinal involvement [4].

Inflammation is strongly involved in the development of complications in cardiovascular diseases. High levels of inflammatory markers have been shown to have a predictive role in future cardiovascular events. For instance, after an acute myocardial infarction, instant activation of multiple local processes begins, resulting in a release of reactive oxygen species and cytokines. The neutrophils and monocytes migrate to the damaged tissue, contributing to the initiation of acute myocardial injury [5]. Elevated levels of inflammatory cytokines are found in patients with HF, and inflammatory processes after organ transplantation may lead to acute allograft rejection, diminution of transplant tolerance, and chronic allograft rejection [6,7].

Neutrophils represent the majority of white blood cells in humans, being involved in the immune response. Depending on the local microenvironment, these cells can present high phenotypic plasticity. As a response to inflammation, neutrophils rapidly migrate to the damaged area [8]. It is known that neutrophils facilitate monocyte differentiation and macrophage polarization. Monocyte recruitment is assisted by neutrophils, and tissue-resident macrophages contribute to neutrophil recruitment [9]. Bloodstream monocytes are the precursors of dendritic cells and tissue macrophages. This subtype of white blood cells is recruited in various infectious diseases, also with key involvement in the systemic inflammatory response, posing a fundamental role in the pathogenesis of an aseptic inflammatory state. Atherosclerosis development and progression can be related to specific monocyte chemokine receptors [10].

Ratios using neutrophil and monocyte count, as well as other ratios of hematological counts, such as white cell, lymphocyte, and platelet counts, might provide additional information on the appearance and progression of complications in multiple diseases.

We included two trials, Akan OY and Yuan C studies, which investigated the prognostic role in two different diseases. They used multiple inexpensive markers that can be determined in the routine blood analysis preoperatively in order to anticipate postoperative complications [11,12].

Akan OY and Bilgir O previously studied markers, such as NMR (neutrophil-to-monocyte ratio), NLR (neutrophil-to-lymphocyte ratio), and NPR (neutrophil-to-platelet ratio) as prognostic factors in patients diagnosed with COVID-19 admitted to the intensive care units. Estimated cut-off values of NMR, NLR, and NPR of 8.9, 2.9, respectively 0.018 were found with prognostic significance in patients who needed intensive care [11].

Yuan C et al. included 1466 patients diagnosed with non-small cell lung cancer considering pretreatment cut-off values of 0.55 for NWR (neutrophil-to-white cells ratio), 0.35 for MLR (monocyte-to-lymphocyte ratio), 204.00 for PLR (platelets-to-lymphocyte ratio), 2.06 for NLR, 0.09 for MWR (monocyte-to-white cells ratio), 0.28 for LWR (lymphocyte-to-white cells ratio). Elevated NLR, NWR, MLR, PLR, MWR, and LWR values were observed to be associated with poor overall survival, although only NWR and MLR were independent prognostic factors [12].

Of equal importance to discovering new biomarkers, which may predict the outcome and evolution of a patient who underwent cardiac transplantation, is identifying those markers which have a low cost, high sensitivity, and are easy to reproduce.

The aim of our study was to investigate the role of NMR, NLR, NPR, NWR, MLR, PLR, MWR, and LWR at the previously studied cut-off values in predicting complications after a heart transplant. We compared high values of these markers to the presence of complications, regardless of cause, post-operative newly diagnosed type 2 diabetes mellitus (DM), documented paroxysmal episode of atrial fibrillation (AFib), acute rejection, and infections within 2 months after surgery. Thus, a significant result of this study may add an important factor in the algorithm of cardiac transplantation evaluation through a new cost-efficient biomarker.

## 2. Materials and Methods

**Figure 1:**
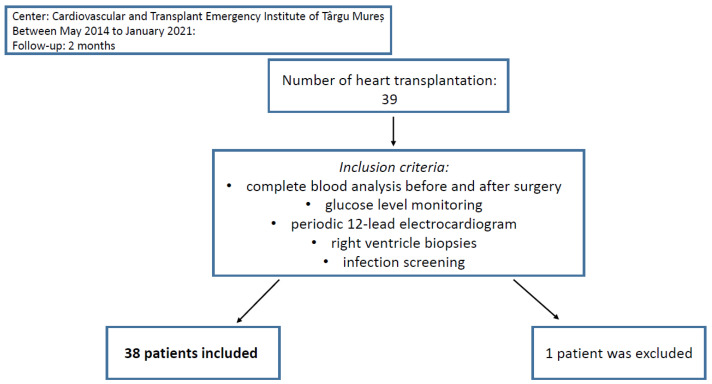
Patient selection and follow-up.

From May 2014 to January 2021, heart transplantation was performed on 39 patients in the Cardiovascular and Transplant Emergency Institute of Târgu Mureș. One patient was excluded from the study because of insufficient data evidence. Informed consent has been obtained from the participants involved (Figure 1).

NMR was calculated as the neutrophil count divided by monocyte count, NLR as the neutrophil count divided by lymphocyte count, NPR as neutrophil count divided by platelet count, NWR as neutrophil count divided by white cells count, MLR as monocyte count divided by lymphocyte count, PLR as platelet count divided by lymphocyte count, MWR as monocyte divided by white cells count, and LWR as the lymphocyte count divided by white cells count. Institutional data sharing prior to the initiation of the study was obtained.

The research protocol was approved by the ethics committee at the Cardiovascular and Transplant Emergency Institute of Târgu Mureș, and the study was conducted in accordance with the Helsinki Declaration.

### 2.1. Management & Follow-Up

All patients included in the study had two sets of complete blood analyses, collected <24 h previous to heart transplantation, respectively, right after as final step of the postoperative protocol. Moreover, glucose level monitoring, periodic 12-lead electrocardiogram, right ventricle biopsies, and infection screening were obtained. C-Reactive Protein (CRP) levels were also registered. We analyzed the levels of NMR, NLR, NPR, NWR, MLR, PLR, MWR, and LWR before and after the heart transplant and the correlation between preoperative biomarkers with the apparition of complications in the first two months after surgery (Figure 1).

Cut-off values were acquired from Akan OY and Yuan C et al. studies. Both trials showed the prognostic role of these markers in patients diagnosed with COVID-19 infection admitted to the intensive care units, respectively, in non-small cell lung cancer [11,12].

### 2.2. Data Processing

Using GraphPad Prism version 9 for quantitative data, we determined the values of markers medians, 25th–75th percentile, maximum and minimum values. The normality test was performed with the Shapiro-Wilks test [13], and we compared values before and after heart transplantation, using *t* test for parametric data and the Wilcoxon test for nonparametric data. Using logistic regression, we analyzed the correlation of preoperative elevated marker values with the apparition of complication of any cause, post-operative newly diagnosed type 2 DM, development of documented paroxysmal episode of AFib, acute rejection, and infections. The significant threshold was set to 0.05.

## 3. Results

Of 38 patients included in the study, four of them were females (10.53%), and 34 were males (89.5%). The youngest patient had 10 years old at the time of the transplant, and the oldest was 61, with a mean age of 41.21 (SD = 13.71), respectively an average body mass of 23.81 (SD = 5.18). The main indication in our cohort was represented by non-ischemic cardiomyopathy, with a proportion of 47.37%, followed by ischemic heart disease in 21.05% of the cases.

The mean ejection fraction prior to the heart transplant was 26.54 (SD = 13.23) %, with an average pulmonary artery pressure of 52.08 (SD = 15.70) mmHg and mean size of the left ventricle of 69.46 (SD = 13.80). Mean values of the primary hematological markers, such as white blood cell (×109/L), neutrophil (×109/L), lymphocyte (×109/L), monocyte (×109/L), and platelet (×109/L) counts were 8.99 (SD = 4.26), 6.51 (SD = 4.17), 1.61 (SD = 0.76), 0.77 (SD = 0.34), respectively 202.11 (SD = 62.61). Standard lipid profile highlights a mean value of total cholesterol of 164.74 (SD = 44.94) mg/dL, with mean levels of low-density lipoprotein cholesterol of 106.05 (SD = 33.52) mg/dL, respectively 36.47 (SD = 10.43) mg/dL for high-density lipoprotein cholesterol. The prior percutaneous coronary intervention was seen in four patients (10.53%) and prior cardiac surgery in five (13.15%). Of these cardiac surgeries, four (10.53%) of them involved heart valve replacements and one involved coronary artery bypass graft surgery (2.63%). Donors’ mean age was 31.39 (SD = 10.67), 68.4% being males and 31.6% females. Gender mismatch has been found in 14 cases (36.8%), three cases occurring in female receipts, and 11 in male receipts (Table 1).

Increased postoperative median values (25th–75th percentile) were seen in pre-CRP 0.43 (0.24–0.88) versus post-CRP 6.37 (4.19–11.69), pre-NMR 8.23 (5.97–9.82) versus post-NMR 17.54 (10.92–23.11), pre-NLR 3.39 (2.38–5.89) versus post-NLR 21.54 (13.74–35.58), pre-NPR 0.027 (0.021–0.036) versus post-NPR 0.112 (0.087–0.155), pre-NWR 0.69 (0.64–0.79) versus post-NWR 0.89 (0.87–0.92), pre-MLR 0.46 (0.33–0.76) versus post-MLR 1.33 (0.07–2.08), pre-PLR 124.4 (80.8–221.6) versus post-PLR 224 (110–296.3). The levels of MWR and LWR were postoperative decreased, with median pre-MWR of 0.09 (0.07–0.11) versus post-MWR 0.05 (0.03–0.07), respectively pre-LWR 0.20 (0.12–0.25) versus post-LWR 0.04 (0.02–0.06). For all data, nonparametric testing for paired samples was applied through the Wilcoxon test. Postoperative CRP, NMR, NLR, NPR, NWR, MLR, and PLR values were significantly increased than preoperative values of the parameters. In contrast, the levels of MWR and LWR significantly decreased after the procedure (Table 2).

Associations were made using the same cut-off levels from Akan OY and Yuan C et al. studies [11,12]. There was no statistically significant relationship between NLR, NPR, NWR, MLR, PLR, and LWR with the presence of any complication. NMR greater than 8.9 (OR: 70.71, 95% CI: 3.39–1473.64; *p* = 0.006) was a risk factor for the apparition of post-operative paroxysmal atrial fibrillation. In contrast, a cut-off value of preoperative MWR of 0.09 (OR: 0.04, 95% CI: 0.003–0.58; *p* = 0.0182) represented a protective factor against atrial fibrillation. High MWR was associated with the risk of complications of any cause (OR: 14.74, 95% CI: 1.05–206.59, *p* = 0.0458) (Table 3).

Complications of any cause were observed in 25 of 38 patients included in our study (65.7%), with a mortality rate of 7.89%. Out of those complications, eight patients presented newly diagnosed type 2 DM (21.1%), seven had mild acute graft rejection (18.4%), whilst postoperative infections were present in 19 (50%), with the main pathogen being Staphylococcus aureus in nine subjects (23.6% from a total number of patients, 47.3% of those with infections). We counted a total of six patients (15.8%) who developed paroxysmal AFib episodes with a rapid ventricular response. Out of these cases, four occurred in the first 48 h after surgery, during inotropic support, and spontaneously converted to sinus rhythm. However, two patients presented symptomatic AFib >48 h after heart transplantation, requiring pharmacological cardioversion with Amiodarone.

Other main reported postoperative complications were postoperative acute kidney injury, dialysis, pericardial effusion, and prolonged inotropic usage. We also reported a case of early phase transient left bundle branch block. One patient presented postoperative severe sinus bradycardia and needed temporary cardiac pacing for 4 days. Subsequent further evolution was favorable.

**Figure 2:**
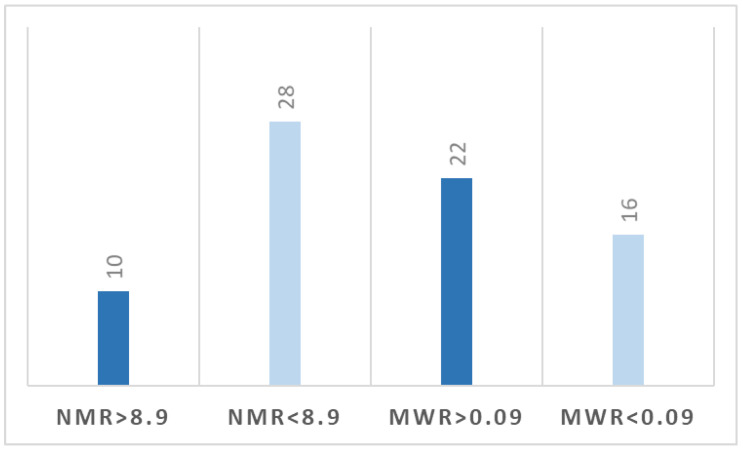
Distribution of patients based on cut-off levels of NMR and MWR.

At the cut-off value of NMR of 8.9, 10 patients (26%) presented preoperative levels above this value, and 28 patients (74%) were below the level. A number of 22 patients (58%) had MWR > 0.09 compared with 16 patients (42%) with MWR < 0.09 (Figure 2).

## 4. Discussion

Systemic inflammation before and after cardiac surgery may be involved in the occurrence of various postoperative complications, thus probably influencing the overall outcome of the patient who underwent heart transplantation. Hematological markers, such as NMR and MWR, may anticipate postoperative complications in various diseases and become prognostic factors that can be easily measured from basic blood analysis with low cost and reproducible parameters. After conducting the statistical analysis of the data collected throughout our research, it was noted that high levels of preoperative MWR were associated with the presence of complications of any cause. The main postoperative complications were newly diagnosed type 2 DM, paroxysmal AFib, mild acute graft rejection, and postoperative infections. Another notable complication that was identified is the need for postoperative dialysis following acute kidney injury, developed in five patients, of whom one patient had persistent renal dysfunction, being discharged with a diagnosis of chronic kidney disease according to the KDIGO guideline. Complications such as newly diagnosed type 2 DM, postoperative infections, and altered renal function can be attributed to the secondary effects of immunosuppressive therapy.

Paroxysmal AFib was documented in six patients, all of them consisting of episodes with a rapid ventricular response. Four of the cases occurred while the patients were under inotropic support in the first 48 h after surgery and spontaneously converted to sinus rhythm. However, over 48 h postoperatively, two patients presented symptomatic AFib episodes in the absence of inotropic support, requiring pharmacological cardioversion with intravenous administration of Amiodarone. It is important to mention the fact that a total of eight patients had permanent AFib prior to heart transplantation. Two patients who presented postoperative AFib were subjects with permanent AFib before surgery. Out of the investigated parameters, an elevated level of preoperative NMR was an independent risk factor in the apparition of paroxysmal AFib, but an increased MWR level played a protective role against this supraventricular arrhythmia. In terms of patients’ distribution based on these cut-off levels, we observed that 26% of the NMR group have been above the threshold, respectively 58% in the MWR group.

Inflammation and the succeeding physio-pathological processes represent part of the initiation and maintenance of AFib. On the other hand, AFib can further support inflammation, thus forming a veritable and dangerous vicious circle with increased morbidity and mortality [14].

There is a fine balance between the beneficial and harmful effects of the monocytes. The beneficial role of monocytes is undeniable when looking at the initial response to pathological changes in cardiac remodeling, although excessive inflammatory response to cardiac insult can be harmful, leading to cardiac fibrosis [15].

Neutrophils play a crucial role in inflammation. A pronounced and prolonged activation is responsible for various cardiovascular diseases. It was identified that elevated levels of NLR are related to new onset AFib, recurrent AFib, and thromboembolic stroke. Considering that neutrophil degranulation affects multiple biological processes, which have been noted to be altered in AFib, this mechanism might be the key to the development of arrhythmia [16,17,18,19].

Because of the stages in which these two types of cells intervene in the inflammation process in a supposed healthy heart after cardiac transplantation, high postoperative levels of neutrophils associated with a decreased number of circulatory monocytes might explain the trigger of the paroxysmal AFib.

Another pro-inflammatory marker, such as CRP, has been reported to be a risk factor for recurrences of AFib. In our study, we observed the fact that CRP values were significantly increased postoperatively, reflecting the elevated level of pro-inflammatory status after heart transplantation. High CRP levels have been associated with AFib recurrences after successful cardioversion [20]. Richter B et al. showed that high-sensitivity C-reactive protein predicted early recurrence of AFib within the first week after catheter ablation but without being involved in long-term ablation outcomes [21].

Elevated levels of CRP in heart transplant receipts are associated with allograft failure. Eisenberg MS, including 99 patients with the cardiac transplant, reported that for every 2-fold elevation of CRP level, there is a 32% increase risk of graft failure [22]. CRP can identify heart-transplants which are at high risk of ischemic events, being associated with the development, severity, and progression of coronary artery disease [23]. High CRP serum concentrations also represent a negative predictive value in renal allograft recipients, being also involved in anticipation of chronic allograft nephropathy and graft failure [24,25].

The prediction value of various biomarkers, such as NLR, has been the aim of various studies, which concluded that high levels of NLR may be associated with severe outcomes in various diseases such as COVID-19 infections, bacterial pneumonia, and malignancies [26,27,28,29]. NLR between 2.3–3.0 is considered to be in a grey area and may represent an early warning of the development of severe maladies such as cancer, atherosclerosis, infection, inflammation, psychiatric disorders, and stress [30].

NLR has been reported as being a predictive factor of Tacrolimus overdose in patients who underwent orthotopic heart transplantation [31]. In liver transplantation receipts, the neutrophil-to-lymphocyte ratio can predict the onset of sepsis [32]. Patients diagnosed with hepatocellular carcinoma who received orthotopic liver transplantation, and presented with elevated levels of NLR, have a higher risk of postoperative tumor recurrence, being associated with increased mortality [33]. Also, in patients with autologous stem-cell transplantation with multiple myeloma, high NLR indicated a poor prognostic factor for progression-free survival and overall survival [34].

Out of the other studied biomarkers, it is important to notice that NMR may serve as a promising inflammatory prognostic tool for patients with pancreatic cancer and locally advanced gastric cancer [35]. In patients diagnosed with severe coronavirus disease 19 (COVID-19) infection, high NMR levels were an independent risk factor, with a sensitivity of 89.47% and a specificity of 80.00% [36].

NWR is associated with poor survival in patients with curatively resected non-small cell lung cancer [12]. Elevated PLR values may indicate a higher risk of fatal stroke occurrence in middle-aged to older patients [37]. High NPR was related to an increased risk of hemorrhagic transformation in patients with acute ischemic stroke, especially in those with parenchymal hematoma [38].

Elevated MLR was significantly associated with stroke-associated pneumonia in acute ischemic stroke patients. Also, MLR is considered to be an independent predictor for the long-term major adverse cardiac event in non-ST-elevation myocardial infarction patients, being independently correlated with the severity of coronary lesions [39,40].

In the case of LWR, lower levels were associated with a decreased risk of mortality in patients with infective endocarditis. In gastric cancer patients, low LWR and high MWR are each predictive of a poor prognosis [41,42].

There are other hematological marker ratios that have been studied previously, for instance, lymphocytes-to-monocytes ratio (LMR). In patients with colorectal cancer, elevated values of LMR, as well as low NLR and low PLR, were associated with longer five-year overall survival [43]. It has been demonstrated that LMR > 2.50 was also significantly associated with improved overall survival and disease-free survival in patients with non-surgically managed small-cell lung cancer [44].

Preoperative inflammatory status, as an independent predictive factor for the postoperative outcome, is one of the most intensely studied fields of research in modern medicine, backed by a large number of research in this regard. The improvement of current therapeutic algorithms is only possible by studying new fields and factors and their predictive value in the context of a complex surgical procedure, such as cardiac transplantation. Considering the rate at which new diagnostic, predictive, and therapeutic algorithms are evolving, it is of utmost importance to maintain the cost of the procedures as low as possible, thus ensuring accessibility and availability to as many distinct hospital units as possible, extending the benefits of the new findings to the maximum number of patients. Various studies aimed to find inexpensive investigations that are already routinely used in diagnostic algorithms and could be used as the foundation on which a revolutionary method could be built. Thus, determining white blood cell, neutrophil, lymphocyte, monocyte, and platelet counts is part of the preoperative process and routine practice in the medical field that could provide the additional foundation for researching new prognostic techniques. Ratios of these markers’ counts have been studied and used as predictive factors in multiple diseases, demonstrating an increased value in predicting negative outcomes and postoperative complications. Even though it is based on highly accessible and repeatable laboratory analysis, the use of these ratios is not widely spread. Cut-off values that are universally applicable need to be determined specifically in cardiac procedures, and in order to do so, additional studies are necessary for the validation of these hematological parameters.

Limitations of our study should be recognized. The absence of data on potential confounding factors, differential losses to follow-up, and information bias can be issued to the retrospective design of the study [45]. Local legislation, the necessity of informed consent, and lack of knowledge on the topic are factors that increase the difficulty of organ harvesting in Romania in the face of an increasing number of patients who would benefit from organ transplantation. This is the reason for the presence of low numbers of cohorts in regional studies [46]. The decreased number of subjects in our cohort may influence the validity of the results by generating false-positive data, thus creating the possibility of overestimating the extent of the association by the misinterpretation of confidence intervals and *p*-values [47]. Another limitation would be asymptomatic, undocumented episodes of AFib. Further prospective studies with larger patient cohorts are needed in order to evaluate the involvement of these markers in postoperative or late-phase complications after a heart transplant and subsequently make drastic conclusions.

## 5. Conclusions

In our study, levels of CRP, NMR, NLR, NPR, NWR, MLR, and PLR were significantly increased postoperative, although MWR and LWR were decreased. Using cut-off values previously studied, we observed that elevated preoperative levels of NMR were associated with the apparition of documented AFib, with high levels of MWR as a protective factor. High MWR was a risk factor in developing complications of any cause in the first two months after heart transplantation.

## Figures and Tables

**Table 1:** Baseline receipts characteristics.

Characteristics	Receipt
**Age (years)** Mean (SD) Min Max	41.21 (13.71) 10 61
**Body Mass Index (kg/m^2^)** Mean (SD) Min Max	23.81 (5.18) 13.2 33.9
**Ejection fraction (%)** Mean (SD) Min Max	26.54 (13.23) 15 65
**Pulmonary artery pressure (mmHg)** Mean (SD) Min Max	52.08 (15.70) 22 82
**Hemoglobin (g/dL)** Mean (SD) Min Max	13.08 (2.44) 8.1 19.0
**White Blood Cell count (×10^9^/L)** Mean (SD) Min Max	8.99 (4.26) 3.47 24.7
**Neutrophil count (×10^9^/L)** Mean (SD) Min Max	6.51 (4.17) 2.09 22.4
**Lymphocyte count (×10^9^/L)** Mean (SD) Min Max	1.61 (0.76) 0.38 3.17
**Monocyte count (×10^9^/L)** Mean (SD) Min Max	0.77 (0.34) 0.17 1.89
**Platelet count (×10^9^/L)** Mean (SD) Min Max	202.11 (62.61) 82 327
**Creatinine level (md/dL)** Mean (SD) Min Max	1.07 (0.32) 0.54 1.82
**Estimated glomerular filtration rate (mL/min/1.73 m^2^)** Mean (SD) Min Max	91.79 (29.06) 45 156
**Total cholesterol level (mg/dL)** Mean (SD) Min Max	164.74 (44.94) 68 269
**Low-density lipoprotein cholesterol (mg/dL)** Mean (SD) Min Max	106.05 (33.52) 42 182
**High-density lipoprotein cholesterol (mg/dL)** Mean (SD) Min Max	36.47 (10.43) 13 59
**Triglycerides level (mg/dL)** Mean (SD) Min Max	112.53 (53.32) 57 281
**Prior percutaneous coronary intervention** Total Percentage (%)	4 10.53%
**Prior cardiac surgery** Total Percentage (%)	5 13.15%
**Heart valve surgery** Total Percentage (%)	4 10.53%
**Donors** Mean age *yrs* (SD) Males (%) Females (%)	31.39 (10.67) 26 (68.4%) 12 (31.6%)
**Gender mismatch** Total (%) Male donor to female receipt (%) Female donor to male receipt (%)	14 (36.8%) 3 (7.9%) 11 (28.9%)

**Table 2:** Comparison between marker values before and after heart transplantation.

**Markers**	**Preoperative**	**Postoperative**	***p* Values**
CRP * Median 25th Percentile 75th Percentile	0.43 0.24 0.88	6.37 4.19 11.69	<0.0001
NMR * Median 25th Percentile 75th Percentile	8.23 5.97 9.82	17.54 10.92 23.11	<0.0001
NLR * Median 25th Percentile 75th Percentile	3.39 2.38 5.89	21.54 13.74 35.58	<0.0001
NPR * Median 25th Percentile 75th Percentile	0.027 0.021 0.036	0.112 0.087 0.155	<0.0001
NWR * Median 25th Percentile 75th Percentile	0.69 0.64 0.79	0.89 0.87 0.92	<0.0001
MLR * Median 25th Percentile 75th Percentile	0.46 0.33 0.76	1.33 0.07 2.08	0.0001
PLR * Median 25th Percentile 75th Percentile	124.4 80.8 221.6	224 110 296.3	0.0268
MWR * Median 25th Percentile 75th Percentile	0.09 0.07 0.11	0.05 0.03 0.07	<0.0001
LWR * Median 25th Percentile 75th Percentile	0.20 0.12 0.25	0.04 0.02 0.06	<0.0001

* Wilcoxon test.

**Table 3:** Association between markers and the presence of postoperative complications.

	Complications ORR/95% CI	Type 2 DM ORR/95% CI	Paroxysmal AFib ORR/95% CI	Acute Rejection ORR/95% CI	Infections ORR/95% CI
**NMR > 8.9**	0.73 0.07–7.46	0.38 0.02–6.89	70.71 3.39–1473.64	0.41 0.01–10.59	0.25 0.01–3.37
**NLR > 2.9**	0.81 0.10–6.14	0.85 0.14–5.18	3.38 0.33–34.58	1.44 0.19–10.53	1.41 0.25–7.82
**NLR > 2.06**	0.67 0.02–16.82	- * -	- * -	0.17 0.007–4.31	3.68 0.11–121.37
**NPR > 0.018**	0.97 0.04–19.96	- * -	- * -	0.82 0.05–11.35	0.52 0.03–6.98
**NWR > 0.55**	- * -	- * -	- * -	- * -	- * -
**MLR > 0.35**	2.80 0.33–23.31	0.75 0.09 -5.67	1.70 0.15–19.10	0.54 0.05–5.01	2.28 0.28–18.67
**PLR > 204**	3.35 0.25–43.66	0.98 0.12–8.08	0.42 0.03–5.18	0 -	1.21 0.20–7.17
**MWR > 0.09**	14.74 1.05–206.59	0.96 0.11–8.10	0.04 0.003–0.58	1.56 0.12–19.97	2.67 0.32–21.97
**LWR > 0.28**	1.56 0.10–23.82	0.66 0.05–7.81	0 -	0.55 0.04–7.07	1.66 0.20–13.24

* cannot be calculated.

## Data Availability

The data presented in this study are available on request from the corresponding author on reasonable request.
